# Popular Fallacies

**Published:** 1870-04

**Authors:** 


### POPULAR FALLACIES.

Allusion was made in a previous number to several very general sentiments as being erroneous in fact, and yet have been received, some of them at least, for generations, as universally acknowledged truths. We wanted to set our readers to thinking for themselves; to get out of the lazy habit of accepting a thing as true, simply on the assertion of some one in whom they repose confidence.

Suppose we were to say that snow was not white; perhaps you would grow indignant at the absurdity of such a proposition, and begin to call names, " foolish," " simple," " no sense," and the like. But if you were asked to prove that snow was white, how many could frame a demonstration? Then, again, when an assertion is made, we should scrutinize the terms, and first understand clearly what is their meaning. It was stated, for example, that

TIGHT LACING

does not tend to produce consumption, but to cure it. We will make a fuller statement.

Tight lacing rather tends to prevent and cure consumption (phthisis) than to cause it. Let us come at the facts by degrees. Women lace; men do not. Do more women die of consumption than men; that is, a sufficiently greater number to authorize the statement that tight lacing must be a prominent cause of the disease in their case?

If not, then there is ground to argue that, as women are so much more confined to the house than men, and yet do not oftener die of consumption, although such confinement tends to cause the disease, it must be that their tight lacing antagonizes causes of consumption.

Those who bend forward, sit crookedly, are very apt to fall into consumption, but every woman will tell you that stays help her to sit erect; and notably, women sit and walk more erectly than men; their stays help them.

Stays compress the lower portion of the chest and lungs; but consumption begins at the top of the lungs, immediately under the collar-bone, and not one time in a thousand is it found in the lower part of the lungs. One would suppose that if lacing injured the lungs, it would be where the lacing was applied, and not at the farthest possible point from it. After our readers have thought these things over, we may say something else. It is no use to point us to the statues of those old women who lived in Greece and Rome, with their waists a mile thick; nobody wants to marry such mountains of fat nowadays, unless there are a million of dollars to balance on the other side. But, with all their rotundity and fat, did they live long? Life averaged then twenty-five, now forty.

### LIFE INSURANCE.

Several years ago we ran a tilt against life insurance companies, and the papers began to call us names. That was a good sign. But epithets are not arguments. Our main argument then was that a main source of profit was in "lapsed legacies;" that is, men, after paying the premiums for several years, got tired of it, or forgot to renew, or were not able to continue it; then all the thousands they paid were lost to them, and went into the insurance coffers without any return. But mark what followed: since then, the more advanced and progressive companies have arranged to do away with this iniquity.

Another point was, that all the hardship and self-denial of paying the premiums fell upon the father of the family, and he was the very person who never received one penny in return; the only compensation being the satisfaction he had that something would be paid to his family if he died.

Then, again, the death of the father had the grief of it lessened by a consideration of dollars and cents in return.

A man came to us in the last stages of consumption. When first married he had his life insured. The premiums were paid for a number of years; meanwhile he became an invalid; every year it was harder and harder for him to pay the premium; but there was his poor family; the last payment was the hardest of all; they sold their only cow, raised the money, sent it to the office the day before the insurance expired; but the office had been removed to another town; the time passed, and he lost all. We asked him why he did not make these representations to the company: "I am dying, and have no money, and am a thousand miles from home."

Reader, what is the use of a man, who has not a dollar, going to law with an insurance company who has millions on hand to fee the ablest lawyers?

We read the other day of an insurance company paying a widow on a policy which had been allowed to lapse. But her husband had been a man of high distinction; the whole community regretted his death — and — yes — and her family connections were among the richest in the country. A cute company that; if we had the name we would publish it gratis. But there is hope; it is now arranged in later companies that lapsed insurances have a specific market value, ascertained from mathematical tables.

Besides this, the best later companies will arrange that a man can pay a certain premium every year, and at his death his family will get the principal; but if he lives a certain number of years, agreed upon, and pays the premium, he shall have a certain amount of money paid to him out and out, or a certain sum paid him every year until he dies. This is a rational and a just and humane arrangement.

Our opinion is, that life insurance, as now conducted, generally is a deception, a cruelty, and a downright fraud on the public; not all life insurance companies, but many of them. Of this, more anon.

### EARLY RISING.

Some years ago we made an onslaught against early rising; it gave one of our Southern exchanges a fit, and he incontinently exclaimed: "Is there no truth on earth?" He never came to.

Suppose we argue the case a little. In the winter the mornings are cold, and who wants to get out of a warm bed and sit on a snow-bank, or shiver for two hours until the house gets warm? We ask, with considerable confidence, Is such a course healthy? Where is the man bold enough to vote "Ay?" Who wants to be boggling about in the dark before day, hunting for slippers and pantaloons, and all that, — his teeth knocking against one another like mad?

And in the cool, delicious summer mornings, when sleep is so heavenly, who ever did get up unless obliged to? And what good did it do him after he was up? His eyes won't open; his brain is asleep, has got no sense, and all he can possibly do is to sit on the side of the bed and think about it. The only people in the world who do want to get up early in the morning are the fellows who were drunk all night, and want water so bad they can't sleep.

Do the French women, who are beautiful at sixty, get up early? We are constantly reading of British noblemen, "bishops and other clergy," dying at seventy, eighty, and ninety years. Is it customary with such to bounce out of bed at daylight?

What can anybody do before breakfast in winter?

We read a good deal of twaddle about the songs of the summer morning birds, and the cool, pure air, and the dew-drops glittering like diamonds on the rose-buds; but they are not diamonds; if they were, it would be worth while getting up early. Suppose the birds do sing, you are so sleepy you don't hear them; now we come jamb up against a wall of adamant which no argument can enter. The out-door air of a summer morning is poisonous to every human creature who breathes it, the most poisonous of all the year. Then what's the use of breathing it? any exercise you take makes you breathe the faster, and consequently the more of the poison. We must not run away with part of a fact; only in whole facts is truth found. George Peabody ate his breakfast habitually at 11 A. M., dinner at 7 P. M., and went to bed at 2 A. M., and lived to seventy years, "healthy, wealthy, and wise."

